# Assessing the Relationships of Expenditure and Health Outcomes in Healthcare Systems: A System Design Approach

**DOI:** 10.3390/healthcare13040352

**Published:** 2025-02-07

**Authors:** Anca Antoaneta Vărzaru

**Affiliations:** Department of Economics, Accounting and International Business, University of Craiova, 200585 Craiova, Romania; anca.varzaru@edu.ucv.ro; Tel.: +40-7-7392-1189

**Keywords:** healthcare systems, healthcare expenditure, healthcare outcomes, system design, healthy life years, health expectancy, standardized death rate, artificial neural networks, predictive models

## Abstract

**Background/Objectives**: The COVID-19 pandemic has significantly altered healthcare systems worldwide, highlighting healthcare expenditure’s critical role in fostering population resilience and wellness. This extraordinary situation has brought to light the delicate balance that governments must maintain between the need to protect public health and budgetary restraints. The relationship between healthcare expenditure and outcomes, such as healthy life years, health expectancy, and standardized death rate, has become a central point in understanding the dynamics of healthcare systems and their capacity to adapt to emerging challenges. **Methods**: Using extensive datasets and predictive approaches such as artificial neural networks, exponential smoothing models, and ARIMA techniques, this study explores these connections in the context of the European Union. **Results**: The study better explains how healthcare financing schemes influence important health outcomes by examining past trends and forecasting future developments. The results show that household healthcare expenditures correlate negatively with standardized death rates and substantially benefit healthy life years and health expectancy. These findings underline the significance of household contributions in influencing health outcomes across various healthcare systems. Long-term and strategic investments in health services are essential, as the pandemic has demonstrated the proactive capacity of well-designed healthcare systems to reduce risks and enhance overall resilience. The results suggest that focused investments can raise life expectancy and lower death rates, supporting the development of robust, adaptable healthcare systems in the post-pandemic era. **Conclusions**: The main contribution of this research is demonstrating the significant role of healthcare expenditure, particularly household contributions, in improving key health outcomes and fostering healthcare system resilience in the EU context.

## 1. Introduction

Over the past few decades, healthcare expenditures have become a key indicator of governments’ commitment to the well-being of their populations. Research indicates that a 1% increase in healthcare expenditure as a percentage of GDP leads to an increase in life expectancy at birth by approximately 0.376 years [1]. This relationship underscores the significant returns on investment in public health, emphasizing the role of adequate funding in improving population health [2].

The COVID-19 pandemic highlighted the critical role of sustainable and efficient healthcare funding. Beyond the absolute amounts spent, the structure and predictability of healthcare financing proved essential in mitigating inequalities in access to care. Nations with robust funding mechanisms faced fewer disparities in healthcare access for vulnerable groups even amidst unprecedented challenges [3].

Efficient healthcare expenditure improves societal well-being and yields long-term economic benefits. Healthy populations are more productive, and the costs associated with managing chronic diseases decrease substantially [4]. For instance, preventive healthcare programs in Nordic countries have significantly reduced the incidence of lifestyle-related diseases, saving billions annually in treatment costs [5]. Therefore, integrating prevention, health education, and infrastructure development into healthcare strategies is crucial for addressing immediate needs and enhancing systemic resilience.

The financial pressures exacerbated by the pandemic have further exposed pre-existing gaps in healthcare systems. Acute shortages of medical personnel, inadequate infrastructure, and inefficient resource allocation have highlighted structural vulnerabilities and amplified healthcare access inequalities [6,7]. However, these issues are not new. Noura [8] emphasized that the deficiencies highlighted by the pandemic had existed long before with the crisis intensifying their impact. Addressing these challenges requires coherent strategies and evidence-based policies to ensure adequate resource utilization and equitable healthcare access [2,7,8].

This study investigates the impact of healthcare expenditure on key indicators such as healthy life years, health expectancy, and standardized death rates both before and after the COVID-19 pandemic. The research provides a differentiated analysis of financing schemes by leveraging artificial neural network analysis and predictive modeling techniques such as ARIMA and exponential smoothing.

The originality of this study lies in its integration of predictive analysis for health outcomes based on historical Eurostat data, emphasizing differences in the effectiveness of various healthcare financing schemes. By exploring the interplay between financial structures and health outcomes in the context of profound economic and social transformations, this research contributes to a deeper understanding of sustainable healthcare funding strategies.

The paper is organized as follows: the first section provides an introduction to the study; the second section details the materials and methods employed; this is followed by the presentation of results, analysis, and discussion; and finally, conclusions and recommendations for future public policy are presented.

## 2. Materials and Methods

### 2.1. Theoretical Background and Hypotheses

#### 2.1.1. Influence of Healthcare Expenditure on Healthcare Outcomes

The relationship between health expenditures and health outcomes, such as death rate and life expectancy, is complex and needs to be better understood [7,8]. While there is a consensus that increased investments in health correlate with higher healthy life expectancy and lower death rates, the mechanisms through which these expenditures directly influence outcomes still need to be explored.

Global studies suggest that countries with higher health expenditures tend to have healthier populations, reporting reductions in preventable disease mortality and improvements in quality of life [8,9,10,11]. However, the relationship between expenditure levels and outcomes is not linear. A larger health budget does not automatically translate into significant improvements, mainly if funds are not used efficiently or distributed equitably. Health expenditures alone are necessary but insufficient to improve health outcomes. Strategic allocation, custom-made to each population’s specific needs, is essential to transform investments into tangible and sustainable benefits for public health [12,13,14,15,16,17,18].

Increasing government expenditure on health in developing countries, particularly those with low and middle incomes, and introducing state regulation in the healthcare sector have been widely advocated [19]. The central argument emphasizes that allocating more public resources to health is vital for improving health conditions and the overall well-being of populations. Higher public health spending reduces disparities in access to medical services, enhances healthcare infrastructure, and supports prevention and education initiatives. Moreover, effective regulation of the healthcare sector plays a crucial role in optimizing resource use and ensuring equitable access to essential services [2,8,10]. Some authors argue that monopolization in purchasing healthcare services can help control costs through economies of scale, reducing inefficiencies and enabling bulk negotiations for better pricing [15,16]. However, others suggest fostering competition among service providers may enhance patient satisfaction, stimulate innovation, and improve service quality [17,18]. Both perspectives highlight the complexity of designing regulatory frameworks that balance cost containment with service quality and accessibility.

Health expenditures remain a determining factor in improving the nation’s overall health [8,19,20]. Governments contribute to enhancing public health through interventions and resource allocations and indirectly through policies that influence the access to and quality of healthcare services. Berger and Messer [21] highlight how changes in public health expenditure can significantly reshape service delivery systems and usage patterns. When governments allocate resources to strengthen healthcare infrastructure, develop preventive programs, and ensure equitable access to quality medical services, the impact becomes evident individually and at the community level.

Moreover, well-directed health expenditures equip countries to respond more effectively to large-scale health crises, such as the COVID-19 pandemic. Robust health systems supported by adequate financial allocations can react swiftly to such events, minimizing their impact on lives and economies [20]. The pandemic demonstrated that countries with well-funded healthcare systems, skilled personnel, infrastructure, and necessary resources managed the crisis more efficiently, reducing mortality rates and adverse economic effects [3,6].

In this context, allocating public resources to healthcare is a moral obligation and an intelligent strategy for ensuring long-term social and economic resilience. Investments in healthcare save lives and lay the foundation for sustainable development by shielding societies from future shocks and reinforcing public trust in the state’s ability to safeguard their well-being [8].

Public funding contributes to providing and optimizing healthcare service consumption. It helps reduce catastrophic health expenditures that could push households into poverty while improving the accessibility and availability of essential services for all citizens regardless of socioeconomic status [22]. Governments must also carefully manage limited budgets to enhance equitable access to healthcare and improve population health [23]. Austerity policies and strict cost-control measures implemented by governments can significantly affect the healthcare sector, influencing financial resources and creating opportunities and challenges for the system [2].

Reducing public expenditures can lead to underfunding essential services, affecting their quality and accessibility. Such policies often exacerbate existing inequalities in access to care, particularly for vulnerable groups. In response to fiscal constraints, governments may turn to alternatives like public–private partnerships or increased user contributions. While these measures can improve efficiency and service variety, they may also deepen reliance on the private sector, raising costs for citizens [24].

Makin and Layton [25] highlight how governments responded to the challenges of the COVID-19 pandemic by adopting highly expansive fiscal policies to increase healthcare expenditure. While these measures were critical in addressing immediate needs, they significantly raised public debt levels, intensifying pressure on fiscal sustainability [2]. Concrete, essential, well-planned measures include optimizing public spending, diversifying tax revenues, and prioritizing strategic health investments. Makina and Layton [25] argue that the effectiveness of these policies hinges on governments’ ability to balance short-term needs with long-term sustainability goals. Fiscal reforms must address current financial pressures and establish a solid framework for resilience against future economic or public health shocks.

Asiskovitch [26] demonstrated that during the COVID-19 pandemic, life expectancy at age 65 was closely linked to public healthcare financing systems. This finding underscores the importance of well-funded and equitable health systems in ensuring longer lives and better quality of life for older populations. Publicly funded healthcare systems provide more consistent support and broader access to medical care for vulnerable groups, which is a key factor during global health crises [27,28,29,30]. A balance between fiscal discipline and sufficient healthcare funding can optimize health outcomes, improve healthy life years, increase health expectancy, and safeguard standardized death rates.

This paper examines the hypothesis that healthcare expenditure schemes significantly influence healthy life years, healthy expectancy and standardized death rates, emphasizing the importance of efficient ways of structuring the healthcare system.

**Hypothesis** **1.** *Increased healthcare expenditure significantly influences healthy life years, health expectancy, and standardized death rates.*

**Figure 1:**
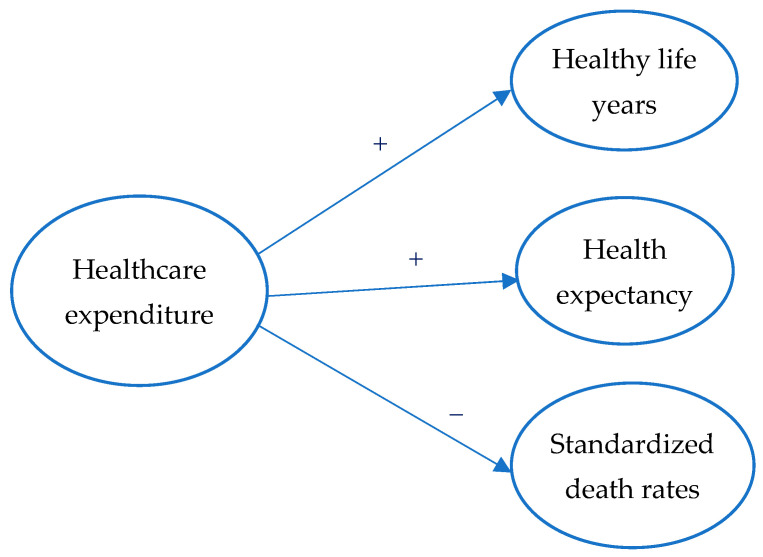
Theoretical model. Source: developed by the author. Note: + positive influences; − negative influences.

Figure 1 graphically illustrates Hypothesis 1, visually representing the proposed relationship between the variables. This figure highlights the underlying connections and pathways that support the hypothesis.

According to this framework, healthcare expenditure is a decisive input that improves health outcomes by enhancing the quality, accessibility, and efficiency of healthcare services. Healthy life years and health expectancy were chosen because they extend beyond traditional mortality metrics to incorporate the quality of life and the duration of time individuals can expect to live in good health. These indicators comprehensively assess healthcare systems’ effectiveness, reflecting preventative and curative care impacts. Furthermore, standardized death rates were included as they capture the broader influence of healthcare interventions on population-wide mortality trends, offering a direct measure of healthcare system performance.

#### 2.1.2. Forecasting Healthy Life Expectancy Based on Healthcare Expenditure Levels

While not all budget allocations immediately or directly enhance population health, well-managed strategic investments in the healthcare sector can yield significant long-term benefits. These investments reduce health risks and build the foundation for healthier societies, fostering broader economic and social well-being [31,32,33].

Two primary approaches dominate the analysis of how government expenditures influence health outcomes. The first draws on Grossman’s model [34,35], which views health as a form of capital that can accumulate or deteriorate over time. The second, proposed by Zweifel and Breyer [36], considers health as an outcome of the healthcare system as a whole. This approach frames health as the product of various inputs, such as public spending, administrative efficiency, and service quality. It highlights the importance of balancing financial investment with the effective allocation of resources to achieve public health goals. Within these frameworks, the efficiency of public health expenditures holds as much significance as the magnitude of expenditure itself [2]. Policymakers face the challenge of increasing health budgets while ensuring these funds are responsibly managed to maximize their impact on public health.

Higher total healthcare expenditure have been strongly linked to a reduction in overall death rates and years of life lost. This fact suggests that increased funding for health systems prevents premature deaths and contributes positively to indicators such as life expectancy and the prevalence of chronic diseases [37]. Moreover, increased health expenditure correlates with lower infant mortality rates, further supporting the argument that investing in healthcare is critical to improving overall health outcomes. This positive effect can be attributed to enhanced access to essential services such as vaccinations, prenatal and postnatal care, and health education programs, particularly in vulnerable communities where economic or geographic barriers often limit access.

The relationship between healthcare expenditure and improved outcomes also underscores the value of effective public policies. The strategic allocation of resources to prevention, healthcare infrastructure, and skilled personnel can amplify the benefits, reducing health inequalities and improving overall societal well-being. Research indicates that countries prioritizing public health expenditure and related social programs often experience substantial improvements in population health, narrowing socioeconomic gaps and fostering social cohesion [38,39].

Every nation must allocate sufficient resources to the health sector to strengthen the link between longevity and economic benefits. Effective healthcare investments have multiplier effects, such as reducing disease burdens, creating healthier and more productive workforces, and lowering future costs associated with chronic illnesses [40,41]. Public funds must be efficiently managed through robust governance and carefully designed policies to optimize resource use and minimize waste.

In addition to strategic spending, countries must establish monitoring and evaluation mechanisms to measure the impact of healthcare investments on development indicators, including reductions in infant mortality, longer life expectancy, and improved quality of life. Success stories in economic literature demonstrate that solid governance and innovative public policies can transform healthcare investments into economic and social progress engines, enhancing financial stability and social cohesion [2].

Health expectancy is a critical global metric for assessing population health. It reflects the average number of healthy years a person can expect, offering insights into the interplay between environmental factors, access to healthcare services, social structures, cultural influences, economic conditions, and genetic predispositions [42,43].

The upward trend in health expectancy observed in recent decades can largely be attributed to improved living standards, diverse lifestyle choices, and expanded educational opportunities [44]. This progress highlights the urgency of addressing current challenges, such as socioeconomic inequalities, pollution, and climate change, which negatively affect health outcomes in many parts of the world.

This study proposes a second hypothesis for forecasting future trends in healthy life years and health expectancy:

**Hypothesis** **2.** *Levels of healthy life years and health expectancy projected based on increased healthcare expenditure growth will be higher than those estimated by continuing historical trends.*

### 2.2. Research Design

This study employs a longitudinal research design to analyze the impact of healthcare expenditure on public health outcomes during the pre- and post-pandemic periods, spanning 2004–2022. The research adopts a quantitative approach to analyze secondary data from Eurostat, covering all 27 European Union member states. The analysis relies explicitly on datasets from the “Health care” and “Health status” sections of Eurostat, ensuring comprehensive and authoritative data for the study. The relationships between healthcare expenditure and public health outcomes were further explored using artificial neural network (ANN) analysis, which provided insights into these connections’ dynamics and assessed their impact.

Predictive modeling techniques were employed, including the ARIMA and Holt and Brown models based on exponential smoothing. These methods facilitated the generation of detailed forecasts regarding the evolution of health outcomes, offering a data-driven perspective on future trends. The results were then interpreted within the socioeconomic context of the European Union with particular attention to contrasts between the pre- and post-pandemic periods. An SEM model was used to assess the extent to which multicollinearity could impact the robustness of the results in the context of these methods. In the case of using artificial neural networks (ANNs), these models have the advantage of being less affected by multicollinearity due to their nonlinear and adaptive nature.

The combination of these methods provides a comprehensive analytical framework. The predictive capabilities of ARIMA, Holt and Brown models complement the nonlinear modeling strengths of ANNs, offering an integrative view of historical trends and future possibilities. SEM further strengthens the analysis by elucidating the structural relationships between variables, ensuring robust results, and testing multicollinearity for ARIMA, Holt, and Brown variables. The study captures the intricate dynamics between healthcare expenditure and outcomes by employing these methods, offering theoretically informed and practically relevant insights. Using multiple methodologies ensures that the research findings are statistically robust and applicable across diverse socioeconomic contexts within the European Union.

The data analysis in this study was conducted using statistical software to ensure robust and reliable results. For artificial neural network (ANN) models and forecasting analyses, SPSS v27.0 was employed. The structural equation modeling (SEM) was performed using SmartPLS v3.0, enabling the examination of complex relationships between variables with high precision and efficiency.

### 2.3. Selected Variables

This research carefully selected variables to comprehensively capture the relationship between healthcare expenditure and public health outcomes, considering various dimensions of investments and their impact on population health. The variables include measures of overall healthcare expenditure and their specific components, alongside essential population health outcomes that reflect health across different life stages. Table 1 shows the research variables.

Total healthcare expenditure (HCE_AS) represents the aggregated investments in healthcare within a country, regardless of funding source. This variable offers a broad overview of financial efforts to sustain healthcare systems. Public contributions, captured through HCE_GCS, highlight the role of government schemes and mandatory contributions in ensuring access to healthcare services. Meanwhile, HCE_VS reflects private sector and voluntary insurance contributions, illustrating how individuals rely on alternative mechanisms to cover healthcare needs. Out-of-pocket payments (HCE_OPP) measure direct household expenditures, highlighting the population’s financial burden and the healthcare system’s equity.

Health outcomes focus on key dimensions of quality of life and mortality. Healthy life years at birth (HLYB) and at age 65 (HLY65) reflect the duration of life free from severe health problems or disabilities, providing insights into both the active years of life and quality of life in old age. Healthy life expectancy at birth (HLEB) and at age 65 (HLE65) combines life expectancy with health status to offer a nuanced perspective on the years lived in optimal health conditions. These indicators are critical for evaluating the impact of healthcare investments on overall well-being.

The standardized death rate (SDR) measures mortality adjusted for the population’s demographic structure [48]. Its formula is expressed as follows (1):

(1) $$SDR=\frac{\sum_{i=1}^{n}\left(d_{i}+w_{i}\right)}{\sum_{i=1}^{n}w_{i}}$$

$d_{i}$—the age-specific death rate for age group i;

$w_{i}$—standard population weight for age group iii (e.g., the population size in that age group in a standard population);

*n*—total number of age groups.

Table 2 shows the descriptive statistics of selected data.

The skewness values for most variables suggest a mild rightward skew, particularly for HCE_VS (1.724), indicating the presence of outliers or countries with exceptionally high healthcare expenditures (voluntary healthcare payment schemes). The kurtosis values for most variables show relatively platykurtic distributions, except for SDR, which exhibits a leptokurtic distribution (8.601), reflecting a higher concentration of values around the mean. The health outcomes, such as HLYB and HLEB, show a more balanced distribution, with mean values around 61–73 years and moderate deviations. The data indicate a mix of normal and non-normal distributions, with some variables exhibiting more extreme values, particularly in healthcare expenditure (voluntary healthcare payment schemes) and mortality-related outcomes.

### 2.4. Research Methods

The study employed various statistical and modeling methods to analyze the data and generate forecasts, capturing the complex relationships between healthcare expenditure and public health outcomes. Artificial neural networks, an advanced machine learning technique capable of modeling often nonlinear relationships between variables, were central to the analysis [49]. By learning and adapting based on patterns in historical data, these networks proved essential in identifying determinants of variations in population health. The multilayer perceptron (MLP) model, based on back-propagation, was chosen to determine these influences, as shown in the following Equation (2):

(2) $$y=(\sum_{i=1}^{n}w_{i}x_{i}+b)=\varphi (W^{T}X+b)$$

*w*, *x*—vectors of weights and inputs;

*b*—bias;

*i*—cases;

*φ*—activation functions.

As activation functions, we used a hyperbolic tangent function (3):

(3) $$f\left(n\right)=\frac{1}{1+e^{-n}}$$

*n*—input variables;

*f*(*n*)—output variables.

Complementing this method, Holt’s exponential smoothing method was applied to analyze and forecast trends. This approach efficiently evaluated current indicator levels and medium-term rates of change. Its forecasting Equation (4), along with smoothing equations for level (5) and trend (6), is as follows [50]:

(4) $${\hat{y}}_{t+\left.h\right|t}=l_{t}+hb_{t}$$

(5) $$l_{t}={\alpha y}_{t}+(1-\alpha )(l_{t-1}+b_{t-1})$$

(6) $$b_{t}={\beta (l}_{t}-l_{t-1})+(1-\beta )b_{t-1}$$

$l_{t}$—an estimate of the level of the series at time *t*; 

$b_{t}$—an estimate of the trend (slope) of the series at time *t*; 

α—the smoothing parameter for the level;

*β*—the smoothing parameter for the trend;

Brown’s exponential smoothing method further enhanced forecast precision by minimizing seasonal fluctuations in the data. This technique captured subtle dynamics within time series, yielding more accurate and context-specific predictions [51]. This method includes the level (7), the trend (8), and the forecast for the next step (9).

(7) $$S_{t}=\alpha y_{t}+(1-\alpha )(S_{t-1}+b_{t-1})$$

(8) $$b_{t}={\beta (S}_{t}-S_{t-1})+(1-\beta )b_{t-1}\;$$

(9) $$F_{t+m}=S_{t}+mb_{t}$$

$y_{t}$—the observed value at time *t*; 

$S_{t}$—the smoothed value for the level at time *t*;

$b_{t}$—the estimated trend at time *t*; 

α—the smoothing parameter for the level;

β—the smoothing parameter for the trend;

$F_{t+m}$—the forecasted value for m steps ahead of time *t*.

The ARIMA (autoregressive integrated moving average) model provides a rigorous time series analysis, delivering robust and statistically grounded forecasts [52]. This model was chosen for its ability to combine autoregressive and moving average components, effectively capturing the dynamic relationships among the variables. The ARIMA formula is as follows (10):

(10) $$\left(1-\sum_{i=1}^{p}\varphi_{i}L^{i}\right){\left(1-L\right)}^{d}X_{t}=\left(1+\sum_{i=1}^{q}\theta_{i}L^{i}\right)\varepsilon_{t}$$

$X_{t}$—data series;

L—lag operator;

$\varphi_{i}$—parameters of the autoregressive part of the model;

$\theta_{i}$—parameters of the moving average part;

$\varepsilon_{t}$—error.

An SEM model was employed to evaluate the potential impact of multicollinearity on the robustness of the results within the context of these methods. Structural equation modeling (SEM) is the appropriate method because it examines the complex relationships among multiple variables and evaluates the factors’ direct and indirect impacts on the final variables [53]. In the context of testing multicollinearity among the three variables—HLYB (healthy life years at birth), HLEB (health expectancy at birth), and HCE_AS (healthcare expenditure—all financing Schemes), SEM provides a robust framework for understanding the interactions and dependencies between them.

Each method was applied integrated, leveraging each technique’s strengths to address the research’s objectives. This combined approach enabled the analysis of current relationships between variables and allowed for the projection of future trends in health outcomes.

## 3. Results

We tested Hypothesis H1 using artificial neural network analysis with a multilayer perceptron (MLP) model, which provided insights into the intricate relationships between healthcare expenditure and public health outcomes. The MLP model facilitated an in-depth exploration of how different types of health-related expenditure contribute to outcomes such as healthy life years, health expectancy, and the standardized death rate. The independent variables included total healthcare expenditure (HCE_AS), expenditures funded through government schemes (HCE_GCS), voluntary schemes (HCE_VS), and out-of-pocket payments by households (HCE_OPP). These variables were analyzed concerning public health outcomes such as healthy life years at birth and age 65, health expectancy at birth and age 65, and standardized death rate. Between the input layer, containing the independent variables, and the output layer, containing the dependent variables, the model incorporated a hidden layer representing the efficiency of healthcare expenditure. The hidden and output layers employed sigmoid activation functions.

**Figure 2:**
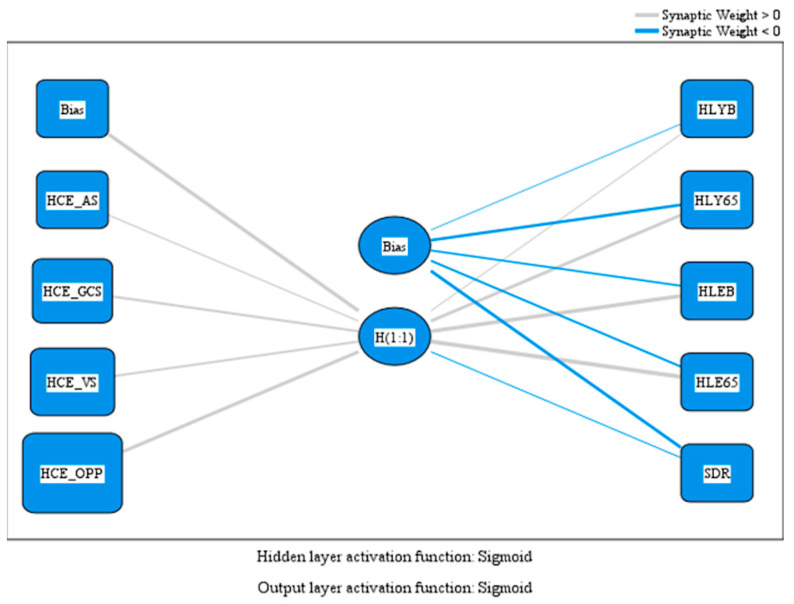
MLP model. Source: authors’ design using SPSS v27.0 (IBM Corporation, Armonk, NY, USA).

Figure 2 illustrates the relationships between healthcare expenditure, healthy life years, health expectancy, and standardized death rate.

Table 3 shows the predicted values of the model variables within the perceptron.

The results reveal both direct and indirect effects of healthcare expenditure on health outcomes via the hidden layer. Each input variable was weighted according to its relevance to the model, as reflected in the values assigned to various neural connections. Out-of-pocket payments (HCE_OPP) demonstrated the highest weight at the input layer, with a coefficient of 1.816, underscoring their significant contribution to the predictive process. Similarly, expenditures through government schemes (HCE_GCS) and voluntary schemes (HCE_VS) also played important roles, albeit less prominently than household payments.

Investments in healthcare, mainly through out-of-pocket payments (HCE_OPP) and government schemes (HCE_GCS), have demonstrated a consistently positive impact on healthy life years at birth (HLYB), supported by a significant baseline bias (1.825). The data further reinforce this trend for individuals aged 65 (HLY65), with substantial contributions from the hidden layer (1.799), highlighting that health investments provide enduring benefits, especially for older populations.

Health expectancy at birth and age 65 also displayed strong positive coefficients with values of 2.417 for HLEB and 2.445 for HLE65. These findings suggest that increased healthcare resources not only extend life expectancy but also enhance its quality. In contrast, the standardized death rate revealed a negative correlation with the hidden layer (−0.715), indicating that higher healthcare expenditure contributes to reduced mortality, underscoring their critical role in mitigating disease burdens.

**Figure 3:**
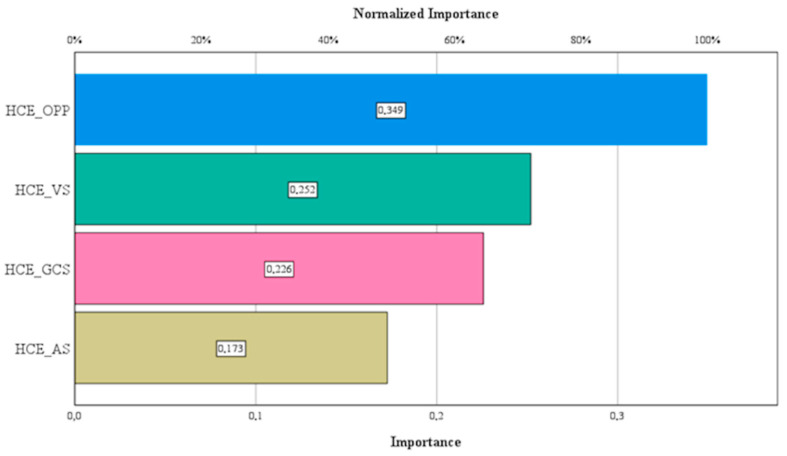
Absolute and normalized importance within the model. Source: authors’ design using SPSS v27.0 (IBM Corporation, Armonk, NY, USA).

Figure 3 visually represents the absolute and normalized importance of the model variables.

Based on normalized values, the analysis identifies household out-of-pocket payments (HCE_OPP) as the most influential factor with a normalized importance of 100%. This influence is followed by voluntary payment schemes (HCE_VS) at 72.1% and government schemes (HCE_GCS) at 64.6%. These findings emphasize the pivotal role of diverse healthcare financing mechanisms in improving health outcomes.

Overall, the findings strongly support Hypothesis H1. Healthcare expenditure significantly impacted healthy life years and health expectancy, reflecting better quality of life and improved public health outcomes. The results highlighted a positive but nuanced influence on the health system’s ability to reduce mortality. This analysis provides empirical evidence reinforcing the role of healthcare investments as a key driver for improving population health outcomes. These conclusions are valuable for formulating evidence-based public policies prioritizing sustainable and equitable healthcare funding.

Hypothesis H2 explores the long-term impact of healthcare expenditure on health outcomes using Holt, Brown, and ARIMA models [50,51,52].

**Figure 4:**
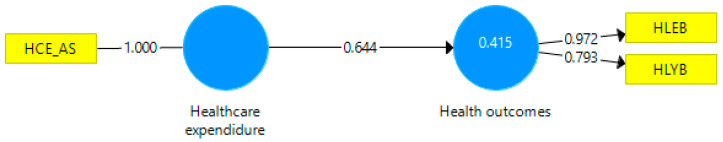
SEM model. Source: authors’ design using SmartPLS v3.0 (SmartPLS GmbH, Bönningstedt, Germany).

To test the multicollinearity of variables included in the context of Holt, Brown, and ARIMA models, structural equation modeling (SEM) was employed. The SEM model incorporates three key variables: HLYB (healthy life years at birth), HLEB (health expectancy at birth), and HCE_AS (healthcare expenditure—all financing schemes), which are also included in the forecasting models. Figure 4 illustrates the SEM model, visually representing the relationships among these variables.

The model’s reliability and validity exposed the indicators presented in Table 4.

The average variance extracted (AVE) of 0.787 confirms convergent validity, as it exceeds the recommended threshold of 0.5. A composite reliability (CR) of 0.880 indicates a high level of internal consistency among the latent constructs. Cronbach’s alpha of 0.772 further demonstrates the reliability of the model’s measurement instruments. Moreover, the path coefficient for the relationship between healthcare expenditure and health outcomes is 0.644, with a T-statistic of 25.599 and a *p*-value of 0.000, indicating a strong and statistically significant positive association.

The SRMR (standardized root mean square residual) in the SEM model is 0.079. This value indicates a good fit for the model, as SRMR values below 0.08 are generally considered acceptable. A lower SRMR value suggests that the differences between the observed and predicted covariance matrices are slight, which means the model represents the data well [53].

Multicollinearity analysis, detailed in Table 5, shows variance inflation factor (VIF) values.

These values are below the commonly accepted threshold of 5, suggesting that multicollinearity is not a concern in this model [53].

The results of the SEM analysis confirm the robustness of the model with high reliability and validity indicators. The low VIF values in Table 4 further support the model’s stability, ensuring that the relationships observed are not biased by multicollinearity among the variables. This comprehensive approach strengthens the predictive power and interpretability of the SEM model in the context of forecasting and causal analysis.

The forecasting model based on Holt’s exponential smoothing method was applied to estimate the evolution of healthy life years at birth (HLYB) using historical data from 2004 to 2022. Several models were tested, and the Holt model demonstrated excellent significance (*p* < 0.001). This method captures long-term trends and recent variations, offering a detailed view of this demographic indicator progression (Table 6).

**Figure 5:**
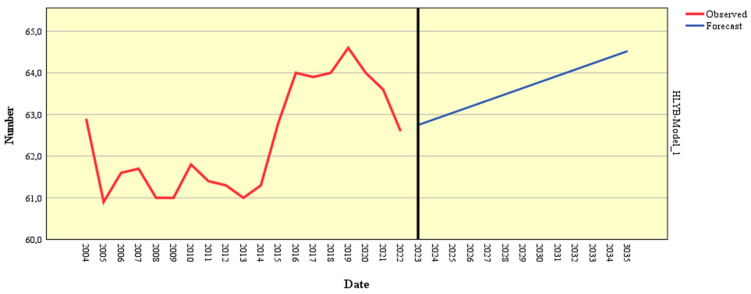
Forecast of HLYB using the Holt model. Source: author’s design using SPSS v.27 (IBM Corporation, Armonk, NY, USA).

The model parameters indicate an alpha (level) value of 1.000, reflecting the model’s emphasis on recent observations when estimating future trends. This result suggests that recent dynamics of the HLYB indicator, shaped significantly by the COVID-19 pandemic, play a critical role in the projections. The high statistical significance (*p* = 0.000) confirms the parameter’s reliability and the robustness of the model. Figure 5 and Table A1 (in the Appendix A) provide a comprehensive forecast breakdown for HLYB’s trajectory.

Historical data reveal a fluctuating pattern in the HLYB indicator between 2004 and 2022 with a notable decline during the pandemic (2020–2022). For instance, after peaking at 64.6 years in 2019, the indicator fell to 62.6 years in 2022, likely reflecting the adverse effects of the COVID-19 crisis on quality of life.

Projections for 2023–2035 indicate a gradual recovery in the HLYB indicator. The trend suggests an increase from 62.7 years in 2023 to 64.5 years in 2035. This result reflects a slow return to pre-pandemic levels, emphasizing that public health measures and healthcare investments will improve quality of life in the coming years. By 2030, HLYB is projected to reach 63.8 years, and by 2035, it is expected to climb to 64.5 years, signifying a steady but modest growth.

The Brown forecasting model, applied to predict the evolution of health expectancy at birth (HLEB), offered a comprehensive analysis of historical trends and future projections. Several models were tested, and the Brown model demonstrated excellent significance (*p* < 0.001). Historical data (2004–2022) point to a gradual increase in HLEB, reflecting consistent improvements in population health (Table 7).

The model’s alpha parameter, estimated at 0.593, reflects a balance between sensitivity to recent fluctuations and the ability to capture long-term trends. Its high statistical significance (t = 5.864, *p* < 0.001) confirms the model’s reliability and the robustness of its projections. This parameter indicates that the model moderately weighs recent data while emphasizing underlying trends.

**Figure 6:**
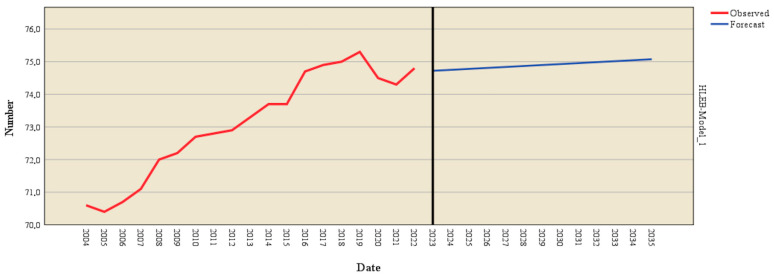
Forecast of HLYB using the Brown model. Source: author’s design using SPSS v.27 (IBM Corporation, Armonk, NY, USA).

Figure 6 and Table A1 in the Appendix A offer a comprehensive overview of the projected expansion of HLEB.

An analysis of historical data shows a steady increase in HLYB from 2004, when it was 70.6 years, to 2019, when it peaked at 75.3 years. However, the negative impact of the COVID-19 pandemic is evident in 2020 and 2021, with HLYB declining to 74.5 and 74.3 years, respectively. By 2022, HLYB rebounded slightly to 74.8 years, signaling potential recovery.

Model forecasts for 2023–2035 suggest a stabilization and gradual rise in HLYB. For instance, the model estimates HLYB at 74.7 years in 2023, climbing incrementally to 75.1 years by 2035. This trend suggests that despite disruptions caused by extraordinary events, structural improvements in public health and quality of life will likely continue to yield positive outcomes.

The ARIMA forecasting model was employed to determine future trends in healthcare expenditure (HCE_AS), leveraging a robust time-series analysis (*p* = 0.002) based on historical data from 2014 to 2022 (Table 8).

According to Table 8, the estimated coefficient for HCE_AS is 128.302, with a standard error of 25.498, indicating precise and stable estimates. The t-value of 5.032 and statistical significance (Sig. = 0.002) confirm the validity of this coefficient with a high degree of confidence, underlining the model’s capacity to deliver accurate forecasts.

**Figure 7:**
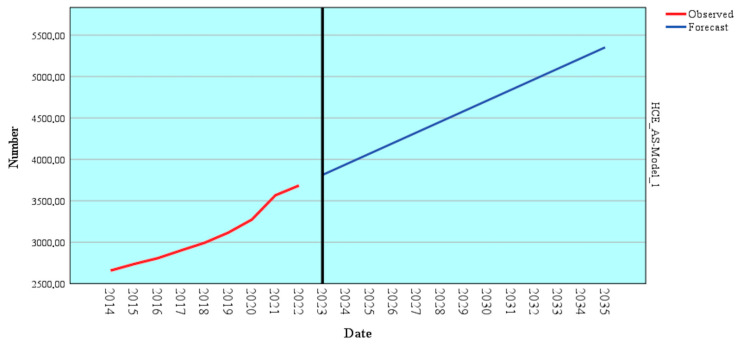
The forecast of HCE_AS depends on the previous annual evolution using the ARIMA model. Source: author’s design using SPSS v.27 (IBM Corporation, Armonk, NY, USA).

Figure 7 and Table A1 in the Appendix A present detailed projections for expanding HCE_AC using ARIMA.

The forecast reveals an upward trajectory in healthcare expenditure over the medium and long term. Projected expenditures rise steadily, reaching 3812.85 in 2023 and climbing to 5352.48 by 2035. This result sustained an upward trend that reflects increasing budgetary allocations to the healthcare sector.

The model also highlights a relatively consistent growth rate over the forecasted period, indicating stability in economic and funding trends for healthcare. The results underscore the need for strategic planning to use allocated funds efficiently.

Two additional ARIMA models were developed to forecast future trends in HLYB and HLEB based on HCE_AS. Several models were tested, and the ARIMA models demonstrated excellent significance (*p* < 0.001). These models, calibrated with data from 2004 to 2022, offer an in-depth perspective on the relationship between healthcare expenditure and health outcomes (Table 9).

The parameters derived from these ARIMA models indicate a strong correlation between financial allocations and health outcomes. For HLYB, the estimated coefficient is 61.777, with a standard error of 3.218 and a t-value of 19.196, showing high statistical significance (*p* = 0.000). This result implies that increased healthcare expenditure substantially impacts healthy life years at birth. Similarly, for HLEB, the estimated coefficient is 72.915, with a standard error of 1.692 and a t-value of 43.090 (*p* = 0.000), further emphasizing the critical role of financial resources in improving health expectancy.

**Figure 8:**
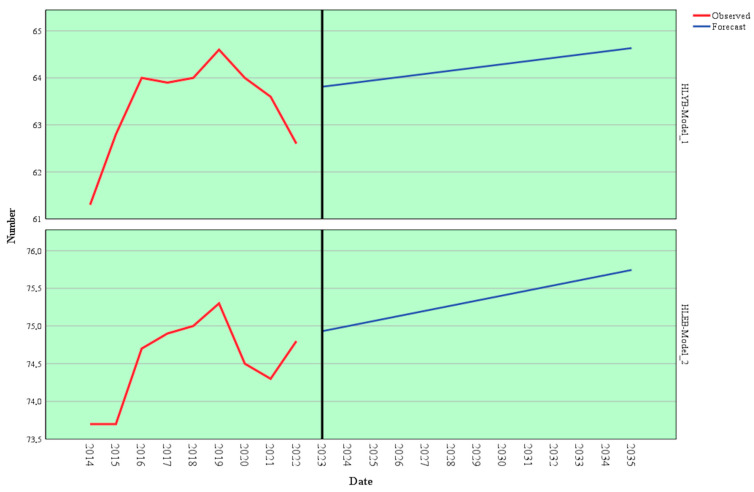
Forecast of HLYB and HLEB depending on the HCE_AS using the ARIMA model. Source: author’s design using SPSS v.27 (IBM Corporation, Armonk, NY, USA).

Figure 8 and Table A1 in the Appendix A provide detailed forecasts for HLYB and HLEB using ARIMA.

Long-term forecasts for these indicators show a positive yet moderate trend. HLYB is projected to start at 63.8 in 2023, gradually increasing to 64.6 by 2035, while HLEB is expected to rise from 74.9 in 2023 to 75.7 by 2035. While this growth is steady, it remains relatively moderate, indicating that while financial allocation is central, its effects are gradual and influenced by factors such as the efficiency of fund utilization, healthcare accessibility, and public policy measures.

These findings underscore the significant potential of healthcare expenditure to enhance population health over the long term. However, they also highlight the decisive role of efficient resource use. The forecasted results suggest that well-designed and effectively implemented healthcare policies can lead to sustained improvements in health outcomes supported by adequate funding and accessible prevention and treatment measures.

**Figure 9:**
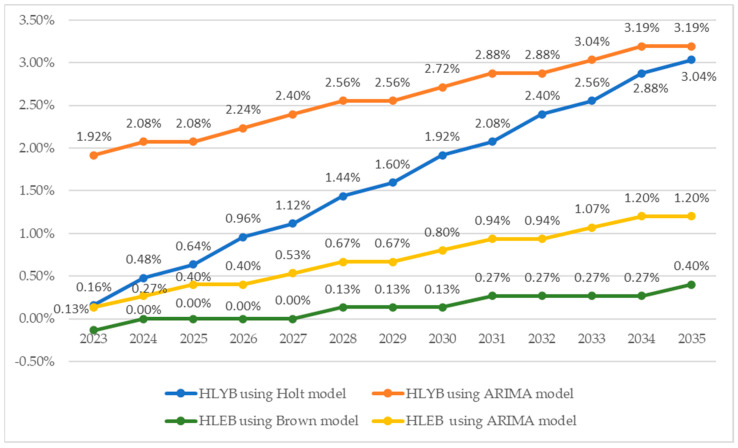
Comparison of the forecasting models. Source: author’s design based on data computed using SPSS v.27 (IBM Corporation, Armonk, NY, USA).

Comparisons between Holt and Brown models (used to forecast HLYB and HLEB based on historical trends) and ARIMA models (forecasting the same indicators using HCE_AS data) reveal notable differences in their growth rates over the long term. HLYB and HLEB show moderate growth trends in the Holt and Brown models. However, ARIMA models, incorporating healthcare expenditure as an independent factor, predict significantly faster and more consistent growth (Figure 9).

For HLYB, the Holt models suggest a steady annual increase of about 0.16% in 2023, gradually accelerating to 3.04% by 2035. In contrast, the ARIMA model, which factors in healthcare expenditure, forecasts a significantly faster growth, starting at 1.92% in 2023 and steadily climbing to 3.19% by 2035. This substantial difference strongly supports hypothesis H2, which suggests that healthy life years and health expectancy levels, projected based on increased healthcare expenditure, are higher than those derived solely from historical trends.

The Brown model offers a cautious perspective on healthy life expectancy at birth (HLEB), forecasting slow and gradual growth. It predicts a slight decline of −0.13% in 2023, with a modest improvement to 0.40% by 2035, suggesting limited progress without significant changes in health policies. In contrast, the ARIMA model is more optimistic, projecting a 0.13% increase in 2023 and a robust annual growth rate of 1.20% by 2035. These projections reinforce Hypothesis H2, emphasizing the transformative potential of additional healthcare investments to improve health expectancy, surpassing historical trends substantially.

## 4. Discussion

Global research highlights that government health expenditures significantly improve public health outcomes, especially in developing nations, by reducing inequalities, enhancing healthcare access, and addressing systemic vulnerabilities through preventive measures and infrastructure development [54,55,56,57,58,59,60].

During pandemic situations, such as the COVID-19 crisis, severe shortages of essential medical equipment, including personal protective equipment, ventilators, and high-flow oxygen systems, exposed significant vulnerabilities [8]. These deficiencies undermined healthcare systems’ capacity to effectively address patient needs, resulting in higher mortality rates and immense stress on healthcare workers. Hospitals often face limited isolation facilities, increasing the risk of virus transmission among medical staff and patients.

Furthermore, the lack of preparedness among healthcare professionals to handle pandemics and insufficient protective equipment and transportation systems further aggravated the crisis. The continuous training of medical staff and establishing efficient patient and personnel transport systems are essential to prevent contamination and ensure a coordinated response in extreme situations. Investments in staff preparedness and resource allocation for patient care and protecting healthcare workers are critical during global health emergencies [61]. Deficiencies in these areas significantly amplified the adverse effects of the pandemic.

Enhanced investments in public health can address these vulnerabilities, enabling more effective crisis responses while improving long-term health outcomes. Thoughtfully planned expenditures can support workforce training, expand infrastructure, and implement policies ensuring equitable access to healthcare services, reducing inequalities in many developing countries [8].

The study proposed two hypotheses to investigate the relationship between healthcare expenditure schemes and three key public health outcomes: healthy life years, health expectancy, and standardized death rates.

The findings from the analysis of Hypothesis H1 confirm a positive and significant influence of health expenditure on indicators such as healthy life years, health expectancy, and standardized mortality rates. These results underscore the importance of consistent investment in the health sector, aligning with trends identified in the literature. Recent studies, such as those by Sultana et al. [8], show that per capita health expenditures positively impact mortality reduction and life expectancy improvement. Better resource allocation toward prevention, primary care, and treatment accessibility for the entire population is decisive for enhancing overall health outcomes. Similarly, this study demonstrates that increasing health budgets has tangible effects on improving public health outcomes.

Moreover, research by Morina et al. [14] reinforces this relationship, showing that a 10% increase in health expenditure per capita correlates with a 3.5-month rise in life expectancy. This finding highlights the substantial influence of health expenditures on population health alongside other factors such as economic development and infrastructure improvements. Evidence from other studies emphasizes that well-targeted public interventions are central to ensuring access to quality healthcare services, especially in rural or disadvantaged areas [62,63]. These findings align with the current study’s conclusions, highlighting the long-term benefits of efficiently directed government expenditure.

However, some voices in the literature question the efficiency of public health expenditures. For instance, studies by Berger and Messer [21] and Sede and Ohemeng [64] suggest that public expenditure only sometimes leads to notable health improvements. From this perspective, individual access to financial resources and quality healthcare services might directly impact health outcomes [65,66]. Nonetheless, these observations do not negate the importance of government intervention but rather point to the need for optimizing resource allocation. This study also highlighted the contributions of household health expenditures, concluding that all funding schemes positively and significantly influence health outcomes.

Research supporting government interventions, such as those by Akinkugbe and Mohanoe [67] or Heijink et al. [68], provides a more comprehensive perspective. These studies demonstrate that adequate public funding reduces avoidable mortality and inequalities in healthcare access, particularly in low- and middle-income countries. Similarly, Novignon et al. [69] found that while private expenditure has a positive impact, public expenditures are essential for ensuring equity in healthcare.

In conclusion, this study’s findings align with the broader body of literature, confirming Hypothesis H1. Increased health expenditure can significantly improve population health by reducing mortality rates, extending life expectancy, and increasing years of good health. These investments become a foundation for enhancing public health and reducing social inequalities within the framework of effective public interventions.

The analysis of Hypothesis H2, which suggests that projected increases in healthcare expenditure lead to higher levels of healthy life years and health expectancy compared to historical trends, yielded results that underscore a significant reality. Findings indicate that sustained increases in health expenditure bolster key public health outcomes and enhance society’s capacity to maintain high levels of well-being over the long term.

These conclusions align with studies by Morina et al. [14], highlighting a direct and positive relationship between healthcare expenditure and health expectancy growth. These findings underscore that well-targeted resources in the healthcare sector contribute to reduced death rates and an extended lifespan in optimal health conditions [70,71]. Similarly, Mohapatra [66] demonstrates that investments in essential services, such as immunization programs and treatments for chronic illnesses, significantly improve population health outcomes. These findings strongly support our hypothesis by emphasizing the role of well-directed public interventions in generating broad benefits for public health.

However, it is essential to consider the broader socioeconomic context. Van den Heuvel and Olaroiu [7] highlight that despite public investments, unequal access to healthcare and socioeconomic disparities can limit the positive effects of health expenditure. Populations with lower incomes or reduced access to medical services remain vulnerable, suggesting that the impact of healthcare expenditure must be understood within an integrated framework of social and economic policies. Similarly, Mosadeghrad [61] stresses the need to incorporate social and economic dimensions into the analysis, pointing out that factors such as education, living conditions, and access to essential resources can amplify or diminish healthcare investments’ effects.

In contrast, our findings partially challenge some more pessimistic perspectives in the literature, arguing that public health expenditures do not always directly impact population health. Studies by Kousar et al. [72] suggest that human capital and population health influence may depend on how efficiently resources are allocated and managed. In this context, health expenditure becomes more effective when complemented by measures to reduce social and economic inequalities and improve healthcare infrastructure [32].

Our results thus support the notion that projected increases in healthcare expenditure have a more substantial impact on health outcomes than mere extrapolations of historical trends. These findings, consistent with the broader literature, underscore the need for an integrated approach that combines increased public funding with complementary measures to address medical but also social, economic, and educational factors [73]. Consequently, Hypothesis H2 is validated, highlighting the importance of coherent and well-coordinated health policies for improving quality of life and ensuring sustainable economic growth.

Healthcare expenditure is a critical indicator of a state’s commitment to the well-being of its citizens. Investments in healthcare infrastructure, advanced medical technologies, and professional training for healthcare workers directly enhance the quality and accessibility of health services [43]. However, the impact is not solely determined by the amount invested but also by how resources are distributed and utilized to meet the actual needs of the population.

Economic growth enables governments to allocate more resources toward building and modernizing healthcare facilities, acquiring state-of-the-art medical equipment, and improving the training of healthcare professionals [74,75]. These investments translate into healthcare services that are not only more accessible but also more efficient in addressing the diverse needs of the population. The development of modern healthcare infrastructure not only improves the immediate health of the population but also stimulates long-term economic growth by reducing healthcare-related costs and creating employment opportunities in the medical sector. An efficient healthcare system directly impacts economic productivity, as a healthy population is better equipped to engage actively in professional and social life. Therefore, prioritizing investments in healthcare infrastructure becomes a strategic imperative for any developing economy aiming to support sustainable health and longevity gains [76,77,78].

### 4.1. Theoretical Implications

The findings of this study offer valuable insights into the interaction between public health expenditure and the performance of healthcare systems, highlighting how public resources can generate positive outcomes in health economic and social terms. Fundamentally, the research emphasizes that the efficiency of allocating these funds is as critical as their absolute level. Targeted investments in healthcare infrastructure, preventive measures, and reducing access inequities can significantly amplify public expenditure’s impact on the quality of life.

In addition to public funding, the study examines the role of out-of-pocket expenditures and compulsory/voluntary healthcare payment schemes. While out-of-pocket payments can increase access in underfunded systems, they often exacerbate inequalities and lead to financial barriers for vulnerable populations. Conversely, depending on their design and implementation, compulsory and voluntary healthcare payment schemes can contribute to more equitable and predictable financing. Integrating these mechanisms into broader health financing strategies is critical to achieving sustainable and inclusive health outcomes.

The COVID-19 pandemic exposed the vulnerabilities of healthcare systems, presenting a timely opportunity to reassess how resources are managed in this sector. The lessons from this challenging period reinforce the urgency of investing in prevention and strengthening healthcare systems’ resilience. When applied to health, resilience theory focuses on a system’s capacity to withstand shocks and adapt swiftly to meet population needs during crises.

This study emphasizes the need for health policies that integrate social and economic dimensions, addressing inequities through custom-made strategies while framing healthcare funding as a strategic investment in equity, resilience, and long-term societal and economic sustainability.

### 4.2. Practical Implications

The research highlights the strategic importance of investing in healthcare infrastructure to improve access, enhance crisis response, and reduce disparities through equitable and well-monitored resource allocation.

Furthermore, the study underscores the impact of financing schemes on healthcare outcomes. Out-of-pocket payments, while providing immediate resources, can result in significant inequities and financial distress for low-income populations. Compulsory health insurance schemes ensure a more stable flow of funds but require careful design to avoid excessive burdens on contributors and to guarantee inclusivity. Voluntary schemes, though flexible, often result in limited coverage and may fail to address systemic inequities unless complemented by strong regulatory frameworks.

Another practical issue arises from the need for a collaborative model between the public and private sectors. The study highlights that healthcare systems relying exclusively on public funding often struggle to address economic and infrastructural challenges. Appropriately regulated private investments can accelerate the adoption of innovative technologies and improve resource efficiency. However, for such collaboration to be effective, authorities must ensure a transparent legislative framework that prevents disparities in access and guarantees equitable treatment for all.

The study highlights the critical need for investing in healthcare infrastructure, focusing on building resilient health systems that can effectively respond to emergencies. This need includes developing a framework for resource allocation, modernizing hospitals, improving emergency care facilities, and expanding digital health systems. A robust system for monitoring and evaluating public fund usage should be implemented, ensuring accountability and transparency through regular audits and performance reviews.

Furthermore, policies for transparent healthcare budgeting should be established to maximize the impact of public funds. Investment in preventive healthcare programs (such as vaccination and screening) is essential to reduce long-term costs and improve population health. Integrating prevention into health planning can lower healthcare costs and improve productivity.

Finally, a collaborative, multi-sectoral approach to policy is necessary with partnerships between the healthcare sector, private industry, academia, and civil society. This approach will help create adaptive and sustainable policies, improving healthcare systems’ resilience and ensuring equitable access to medical care for all populations.

### 4.3. Limitations and Further Research

While offering valuable contributions to understanding the relationship between public health expenditures and healthcare system performance, this research has limitations. First, it excludes GDP and GDP per capita, despite their relevance to healthcare funding, to focus on the direct impact of resource allocation; future research should consider these economic factors for a deeper analysis. Second, demographic variables like the mean age of the population, which significantly affect health indicators such as life expectancy and mortality, were omitted and should be integrated into subsequent studies. Third, lifestyle factors such as smoking, alcohol consumption, and diet, which are key determinants of health outcomes, were not analyzed. Their inclusion in future research could enhance the study’s validity and provide a more comprehensive understanding. While significant correlations between expenditures and health outcomes have been highlighted, the underlying causal mechanisms have yet to be explored in depth. Addressing these mechanisms would require more detailed and interdisciplinary investigations.

Another limitation of this study is the lack of detailed analysis of the structure of healthcare expenditures, such as allocations for medicines, prevention, or infrastructure. While the study focuses on the overall impact of healthcare spending, the specific distribution of these resources likely influences health outcomes significantly. Future research should incorporate data from sources like Eurostat on healthcare spending by category to provide a more granular understanding of how resource allocation impacts public health.

Another key limitation is the exclusion of potential confounding factors such as the mean age of the population, preexisting comorbidities, and the estimated pre-existing quality of life. These variables are often associated with health outcomes and may have moderated or mediated the observed effects. Furthermore, the study does not account for the heterogeneity of healthcare systems across countries, including variations in policy implementation, healthcare access, and cultural factors influencing health-seeking behavior. These aspects could introduce variability in outcomes not addressed in the current analysis.

However, these limitations pave the way for future research directions. Future research should consider incorporating these confounding factors into the analysis, employing more comprehensive datasets that include demographic, epidemiological, and qualitative variables. Such an approach could offer a more nuanced understanding of the mechanisms linking healthcare expenditure with health outcomes and enhance the generalizability of findings across different contexts.

Another area for improvement lies in measuring the efficiency of public health funding. Traditional indicators such as health expectancy or death rate provide an overarching view but often fail to capture nuances related to quality of life or inequalities in access to healthcare services. External factors, such as pandemics or economic crises, further complicate predictive analysis and may distort conclusions, underscoring the need for sensitive approaches.

Subsequent studies could also investigate more profound expenditure efficiency, examining not only their volume but also their allocation across various areas of public health. Investigating regional disparities and the impact of factors such as income levels or education on expenditure efficiency could yield valuable insights for policies more tailored to local needs.

Another promising research direction involves analyzing the integration of private funding into public healthcare systems. Answering this question would require study models incorporating economic data and social perspectives. Considering the lessons learned from the COVID-19 pandemic, future research could explore how healthcare expenditure can enhance system resilience to global crises, accounting for medical dimensions and social and economic stability.

## 5. Conclusions

Under increasingly complex global challenges, from health crises to economic and social pressures, a well-funded and efficiently managed healthcare system is central to ensuring population well-being and long-term stability. This study underscores the fundamental importance of adequate and efficient healthcare expenditure in securing public welfare and economic stability over the long term. The findings demonstrate a clear relationship between public health expenditures and the performance of healthcare systems, which are measured through indicators such as health expectancy and mortality rates. Furthermore, they show that increased financial allocations, coupled with well-targeted policies, can significantly improve quality of life while simultaneously alleviating pressures on economic and social systems.

The COVID-19 pandemic has highlighted the vulnerabilities of many healthcare systems and the urgent need for reforms prioritizing prevention and strengthening health infrastructure. In this context, well-planned public investments in healthcare emerge as a necessity and a critical strategy to prepare medical systems for future crises and support sustainable economic development. Resilience in healthcare systems forms a central part of this equation with the study’s conclusions pointing to the need for responsible and transparent resource management to achieve it.

At the same time, the research shows that more than simply increasing expenditures is needed. Efficiency in using funds, directing them toward strategic priorities, and reducing inequalities in access to healthcare are all aspects that must be integrated into public policies. Particularly in countries with limited resources, balancing public funding with private sector involvement, supported by a well-structured legislative framework, becomes decisive for modernizing and ensuring the sustainability of healthcare systems.

This study contributes to understanding the complex relationship between public health expenditures and outcomes, providing a solid foundation for shaping better-adapted public policies. Its conclusions also highlight the need for continued research in this area to better address the needs of an ever-changing world. Therefore, a well-funded and efficiently managed healthcare system is a priority for the present and a strategic investment in a more equitable, healthy, and prosperous future.

## Figures and Tables

**Table 1:** Research variables.

Variable	Dataset	Measures	References
HCE_AS	Healthcare expenditure—all financing schemes	Euro per inhabitant	[45]
HCE_GCS	Healthcare expenditure—government schemes and compulsory contributory healthcare financing schemes	Euro per inhabitant	[45]
HCE_VS	Healthcare expenditure—voluntary healthcare payment schemes	Euro per inhabitant	[45]
HCE_OPP	Healthcare expenditure—household out-of-pocket payment	Euro per inhabitant	[45]
HLYB	Healthy life years in absolute value at birth	Year	[46]
HLY65	Healthy life years in absolute value at 65	Year	[46]
HLEB	Health expectancy in absolute values at birth	Year	[47]
HLE65	Health expectancy in absolute values at 65	Year	[47]
SDR	Standardized death rate	Rate	[48]

Source: developed by the author based on Eurostat data [45,46,47,48].

**Table 2:** Descriptive statistics.

	N	Minimum	Maximum	Mean	Std. Deviation	Skewness	Kurtosis
HCE_AS	243	380.1	6590.2	2678.275	1732.6539	0.462	−1.225
HCE_GCS	243	264.8	5674.7	2071.226	1486.9339	0.597	−1.054
HCE_VS	243	2.6	718.0	139.458	143.7818	1.724	3.461
HCE_OPP	243	77.2	1023.3	464.668	226.6792	0.092	−1.038
HLYB	243	51.4	73.6	61.965	4.8000	0.384	−0.281
HLY65	243	3.9	16.2	8.876	2.7729	0.310	−0.211
HLEB	243	64.8	80.3	73.565	4.1875	−0.349	−1.203
HLE65	243	8.5	19.5	14.741	2.9840	−0.320	−1.299
SDR	243	790.7	9160.8	1590.514	1436.6893	2.967	8.601

Source: developed by the authors using SPSS v.27.0 (IBM Corporation, Armonk, NY, USA).

**Table 3:** MLP parameters.

		Hidden Layer 1	Output Layer				
		H(1:1)	HLYB	HLY65	HLEB	HLE65	SDR
Input Layer	(Bias)	1.825					
	HCE_AS	1.096					
	HCE_GCS	1.396					
	HCE_VS	1.324					
	HCE_OPP	1.816					
Hidden Layer 1	(Bias)		−0.683	−1.519	−1.099	−1.124	−1.757
	H(1:1)		0.979	1.799	2.417	2.445	−0.715

Source: developed by the authors using SPSS v.27.0 (IBM Corporation, Armonk, NY, USA).

**Table 4:** Reliability, validity and path coefficients.

	Original Sample	Sample Mean	Standard Deviation	T Statistics	*p* Values
Average Variance Extracted	0.787	0.783	0.030	26.482	0.000
Composite Reliability	0.880	0.877	0.020	44.062	0.000
Cronbach’s alpha	0.772	0.769	0.031	24.937	0.000
Path coefficient(Healthcare expenditure → Health outcomes)	0.644	0.645	0.025	25.599	0.000

Source: author’s design using SmartPLS v3.0 (SmartPLS GmbH, Bönningstedt, Germany).

**Table 5:** Variance inflation factor.

	VIF
HCE_AS	1.000
HLEB	1.654
HLYB	1.654

Source: author’s design using SmartPLS v3.0 (SmartPLS GmbH, Bönningstedt, Germany).

**Table 6:** Exponential smoothing model parameters for HLYB forecasting.

Model			Estimate	SE	t	Sig.
HLYB-Model_1	No Transformation	Alpha (Level)	1.000	0.217	4.602	0.000

Source: author’s design using SPSS v.27 (IBM Corporation, Armonk, NY, USA.

**Table 7:** Exponential smoothing model parameters for HLEB forecasting.

Model			Estimate	SE	t	Sig.
HLEB-Model_1	No Transformation	Alpha (Level and Trend)	0.593	0.101	5.864	0.000

Source: author’s design using SPSS v.27 (IBM Corporation, Armonk, NY, USA).

**Table 8:** ARIMA model parameters for HCE_AS.

Model			Estimate	SE	t	Sig.
HCE_AS-Model_1	HCE_AS	No Transformation	128.302	25.498	5.032	0.002

Source: author’s design using SPSS v.27 (IBM Corporation, Armonk, NY, USA).

**Table 9:** ARIMA model parameters for HLYB and HLEB.

Model			Estimate	SE	t	Sig.
HLYB-Model_1	HLYB	No Transformation	61.777	3.218	19.196	0.000
HLEB-Model_2	HLEB	No Transformation	72.915	1.692	43.090	0.000

Source: author’s design using SPSS v.27 (IBM Corporation, Armonk, NY, USA).

## Data Availability

Data are available in a publicly accessible repository. The data presented in this study are openly available: https://ec.europa.eu/eurostat/databrowser/view/hlth_hlye__custom_13873351/default/table?lang=en (accessed on 2 November 2024); https://ec.europa.eu/eurostat/databrowser/view/hlth_sha11_hf__custom_13873147/default/table?lang=en (accessed on 2 November 2024); https://ec.europa.eu/eurostat/databrowser/view/hlth_silc_17__custom_13873518/default/table?lang=en (accessed on 2 November 2024); https://ec.europa.eu/eurostat/databrowser/view/hlth_cd_asdr2__custom_13873618/default/table?lang=en (accessed on 2 November 2024).

## Appendix A

**Table A1:** Historical and future trends.

	Historical Trend			Forecast of HLYB Using Holt Model	Forecast of HLYB Using Brown Model	Forecast of HCE_AS Using ARIMA Model	Forecast of HLYB Using ARIMA Model	Forecast of HLEB Using ARIMA Model
Year	HLYB	HCE_AS	HLEB	Predicted HLYB	Predicted HLEB	Predicted HCE_AS	Predicted HLYB	Predicted HLEB
2004	62.9	-	70.6	-	-	-	-	-
2005	60.9	-	70.4	-	-	-	-	-
2006	61.6	-	70.7	-	-	-	-	-
2007	61.7	-	71.1	-	-	-	-	-
2008	61.0	-	72.0	-	-	-	-	-
2009	61.0	-	72.2	-	-	-	-	-
2010	61.8	-	72.7	-	-	-	-	-
2011	61.4	-	72.8	-	-	-	-	-
2012	61.3	-	72.9	-	-	-	-	-
2013	61.0	-	73.3	-	-	-	-	-
2014	61.3	2658.13	73.7	-	-	-	-	-
2015	62.8	2737.20	73.7	-	-	-	-	-
2016	64.0	2807.27	74.7	-	-	-	-	-
2017	63.9	2902.08	74.9	-	-	-	-	-
2018	64.0	2994.18	75.0	-	-	-	-	-
2019	64.6	3116.25	75.3	-	-	-	-	-
2020	64.0	3273.66	74.5	-	-	-	-	-
2021	63.6	3566.78	74.3	-	-	-	-	-
2022	62.6	3684.55	74.8	-	-	-	-	-
2023	-	-	-	62.7	74.7	3812.85	63.8	74.9
2024	-	-	-	62.9	74.8	3941.15	63.9	75.0
2025	-	-	-	63.0	74.8	4069.46	63.9	75.1
2026	-	-	-	63.2	74.8	4197.76	64.0	75.1
2027	-	-	-	63.3	74.8	4326.06	64.1	75.2
2028	-	-	-	63.5	74.9	4454.36	64.2	75.3
2029	-	-	-	63.6	74.9	4582.67	64.2	75.3
2030	-	-	-	63.8	74.9	4710.97	64.3	75.4
2031	-	-	-	63.9	75.0	4839.27	64.4	75.5
2032	-	-	-	64.1	75.0	4967.57	64.4	75.5
2033	-	-	-	64.2	75.0	5095.88	64.5	75.6
2034	-	-	-	64.4	75.0	5224.18	64.6	75.7
2035	-	-	-	64.5	75.1	5352.48	64.6	75.7
